# Age-Dependent Association Between Cognitive Reserve Proxy and Longitudinal White Matter Microstructure in Older Adults

**DOI:** 10.3389/fpsyg.2022.859826

**Published:** 2022-06-10

**Authors:** Rostislav Brichko, Anja Soldan, Yuxin Zhu, Mei-Cheng Wang, Andreia Faria, Marilyn Albert, Corinne Pettigrew, The BIOCARD Research Team

**Affiliations:** ^1^Department of Neurology, School of Medicine, Johns Hopkins University, Baltimore, MD, United States; ^2^Department of Biostatistics, Bloomberg School of Public Health, Johns Hopkins University, Baltimore, MD, United States; ^3^Department of Radiology, School of Medicine, Johns Hopkins University, Baltimore, MD, United States

**Keywords:** diffusion tensor imaging, cognitive reserve, brain maintenance, aging, white matter microstructure, vascular risk, *APOE*

## Abstract

**Objective:**

This study examined the association of lifetime experiences, measured by a cognitive reserve (CR) composite score composed of years of education, literacy, and vocabulary measures, to level and rate of change in white matter microstructure, as assessed by diffusion tensor imaging (DTI) measures. We also examined whether the relationship between the proxy CR composite score and white matter microstructure was modified by participant age, *APOE*-ε4 genetic status, and level of vascular risk.

**Methods:**

A sample of 192 non-demented (*n* = 166 cognitively normal, *n* = 26 mild cognitive impairment) older adults [mean age = 70.17 (SD = 8.5) years] from the BIOCARD study underwent longitudinal DTI (mean follow-up = 2.5 years, max = 4.7 years). White matter microstructure was quantified by fractional anisotropy (FA) and radial diffusivity (RD) values in global white matter tracts and medial temporal lobe (MTL) white matter tracts.

**Results:**

Using longitudinal linear mixed effect models, we found that FA decreased over time and RD increased over time in both the global and MTL DTI composites, but the rate of change in these DTI measures was not related to level of CR. However, there were significant interactions between the CR composite score and age for global RD in the full sample, and for global FA, global RD, and MTL RD among those with normal cognition. These interactions indicated that among participants with a lower baseline age, higher CR composite scores were associated with higher FA and lower RD values, while among participants with higher age at baseline, higher CR composite scores were associated with lower FA and higher RD values. Furthermore, these relationships were not modified by *APOE*-ε4 genotype or level of vascular risk.

**Conclusion:**

The association between level of CR and DTI measures differs by age, suggesting a possible neuroprotective effect of CR among late middle-aged adults that shifts to a compensatory effect among older adults.

## Introduction

*Cognitive reserve* (CR) is commonly defined as an attribute of the brain that provides for better-than-expected cognitive performance in the presence of age- or disease-related brain changes (Stern et al., 2020; Reserve and Resilience, 2021)^1^. Although the neurobiological mechanisms underlying CR are not yet well understood, previous research suggests that certain lifetime experiences—including increased education, greater occupational complexity, higher literacy, and bilingualism—may reduce the impact of brain injury or disease on cognitive performance (Solé-Padullés et al., 2009; Brickman et al., 2011; Schreiber et al., 2016; Rentz et al., 2017). These lifetime experiences, in combination with genetic factors, are hypothesized to enable the brain to better cope with or compensate for brain aging or disease. The related concept of *brain maintenance* is often used to refer to the relative absence of age-related or disease-related changes in neural resources over time as a determinant of preserved cognitive performance. It is hypothesized that these same lifetime experiences, as well as other factors, may directly prevent or slow age- or disease-related brain changes, thereby reducing cognitive decline and clinical symptoms (Stern et al., 2020).

The present study examines one possible mechanism by which lifetime experiences may promote brain maintenance by investigating how proxies of cognitive reserve (including years of education, literacy, and vocabulary) affect brain white matter microstructural properties over time, as assessed by diffusion tensor imaging (DTI). DTI is a magnetic resonance imaging (MRI) technique for measuring the diffusion of water in tissue. Higher fractional anisotropy (FA), a measure of directional diffusion, and lower radial or mean diffusion (RD and MD, respectively) in white matter tracts are commonly considered to represent greater white matter structural “integrity” (Bennett and Madden, 2014), though their biological interpretability is limited (see Jones et al., 2013). Prior DTI studies have shown that FA decreases with age, whereas RD and MD increase with age, suggesting a decrease in white matter microstructural integrity with advanced age (e.g., Bennett and Madden, 2014; Bender et al., 2016). Of relevance to the current investigation, higher FA and lower RD or MD in white matter tracts have been linked to better cognitive performance among older adults (e.g., Sasson et al., 2013; Bennett and Madden, 2014; Coelho et al., 2021), suggesting that maintenance of white matter microstructural integrity with increasing age would be important for maintaining cognitive performance in aging.

Relatively few studies have investigated how cognitive reserve proxies, such as education, may affect white matter microstructure among middle-aged and older individuals with normal cognition and those with mild cognitive impairment (MCI). To our knowledge, these studies have all been cross-sectional in nature and results have been mixed. For instance, Teipel et al. (2009) found that cognitively normal older individuals with more years of education had higher FA values in medial temporal lobe regions and other cortical and subcortical structures, compared to those with less education. Similarly, Kaup et al. (2018) found that having occupations with greater cognitive complexity was associated with higher FA globally, with strongest effects in frontal and temporal lobes. Additionally, higher socio-economic status (Johnson et al., 2013), greater literacy (Resende et al., 2018), and bilingualism (Luk et al., 2011) were related to higher regional white matter FA among non-demented older adults. Although these studies suggest that greater educational or occupational achievement may have neuroprotective effects on white matter microstructure, other studies examining these relationships reported null results (Casaletto et al., 2020; Neth et al., 2020), or associations in the opposite direction. For example, Vaqué-Alcázar et al. (2017), found that cognitively normal older individuals with higher levels of education had lower FA measures in several white matter tracts. Likewise, higher CR composite scores that incorporated measures of education, occupation, and cognitive leisure activity were associated with lower FA in the corpus collosum among older participants with normal cognition or MCI (Arenaza-Urquijo et al., 2011).

The reason(s) for the discrepancy in results across prior studies remain unclear, but could be related to differences in white matter tracts examined and participant characteristics, including age, proportion of individuals at genetic risk for Alzheimer’s disease (AD), level of vascular risk, and cognitive status (cognitively normal vs. non-demented, which includes cognitively normal and MCI participants). One goal of the current study, therefore, was to systematically investigate whether the relationship between a proxy measure of CR and white matter microstructural integrity is modified by participant age, *APOE*-ε4 genetic status (the main genetic risk factor for late-onset AD; Corder et al., 1993), and level of vascular risk. Additionally, to our knowledge, no prior studies have examined the impact of cognitive reserve proxies on brain white matter microstructure over time. To address these gaps in the literature, the present study uses DTI measures taken at multiple timepoints to examine whether lifetime experiences, measured by a CR composite proxy score, are associated with the level and/or rate of change in white matter microstructure over time (mean DTI follow-up = 2.5 years, max = 4.7 years). White matter microstructure was examined both globally (using the average across 8 major tracts) and within tracts connecting medial temporal lobe (MTL) structures. These tracts were chosen because they have previously been linked to CR proxies, as discussed above. Furthermore, while most prior studies on this topic focused on FA as a measure of white matter microstructure and had relatively small sample sizes (≤100 participants, e.g., Teipel et al., 2009; Arenaza-Urquijo et al., 2011; Luk et al., 2011; Johnson et al., 2013; Vaqué-Alcázar et al., 2017; Resende et al., 2018), the current study assessed both FA and RD and included 192 non-demented participants, including 166 with normal cognition and 26 with MCI.

## Materials and Methods

### Study Design and Participant Selection

Participants included in the current study are part of the ongoing prospective, longitudinal BIOCARD study, which was initiated at the National Institutes of Health (NIH) in 1995. The BIOCARD study has the overarching goal of identifying variables among individuals with normal cognition that could predict the onset of mild to moderate symptoms of AD. After providing written informed consent, 349 cognitively normal, primarily middle-aged participants were enrolled in the study. Approximately 75% of the participants had a family history of AD dementia. While the study was at the NIH, participants completed annual clinical and cognitive assessments, as well as biomarker assessments approximately every other year. The study was stopped in 2005 for administrative reasons and resumed at Johns Hopkins University (JHU) in 2009, upon approval by the JHU Institutional Review Board. Annual blood draws and clinical and cognitive evaluations were reinitiated in 2009. The biennial collection of MRI scans (including DTI) was resumed in 2015. Additional details on the resumption of other biomarker assessments are described in Pettigrew et al. (2020).

This study reports data from 192 non-demented participants with DTI data collected between 2015 and 2020. For the purposes of these analyses, “baseline” was defined as the first available DTI scan. Of note, the MRI scans collected at the NIH did not include DTI sequences, thus the first possible DTI scan in this cohort was acquired in 2015.

### Cognitive and Clinical Assessment

The annual visits at JHU include a neuropsychological battery, clinical assessments, and a semi-structured interview based on the Clinical Dementia Rating Scale (CDR, Morris, 1993; see Albert et al., 2014 for details). The diagnosis of MCI (Albert et al., 2011) and dementia (McKhann et al., 2011) followed recommendations by the National Institutes on Aging and Alzheimer’s Association working group reports. Three types of information were used as the basis for establishing a syndromic diagnosis (i.e., cognitively normal, MCI, impaired not MCI, or dementia): (1) clinical data reporting the individual’s medical, neurological, and psychiatric status; (2) worsened cognitive performance on the neuropsychological battery, based on review of longitudinal testing from multiple domains and comparison to published norms; and (3) reports of changes in cognition by the individual and by collateral sources based on the CDR. Psychiatric, neurological, and medical information collected at the visit were used to identify the likely etiology(ies) of the syndrome for individuals judged to be impaired. When conflicting information from neuropsychological battery performance and the CDR interview were reported (e.g., the collateral source and/or participant reported changes in cognition in daily life, but cognitive testing did not show changes or vice versa), a diagnosis of Impaired not MCI was made (see Albert et al., 2014). Participants with a diagnosis of Impaired not MCI (*N* = 29) were included in the group of cognitively normal participants, but results were comparable when they were excluded from analysis (data not shown). All clinical diagnoses were made independently from knowledge of any biomarker measures.

### MRI Acquisition

MRI scans were acquired on a 3 T Phillips Achieva scanner (Eindhoven, The Netherlands). The multi-modal protocol included magnetization-prepared rapid gradient echo (MPRAGE) scans used for anatomical reference and image registration (TR = 6.8 ms, TE = 3.1 ms, shot interval 3,000 ms, flip angle = 8°, FOV = 240 × 256 mm^2^, 170 slices with 1 × 1 × 1.2 mm^3^ voxels, and scan duration = 5 min 59 s). Diffusion-weighted images were acquired from a spin echo sequence (TR = 7.5 s, TE = 75 ms, FOV = 260 × 260, slice thickness = 2.2 mm, flip angle = 90, b-value = 700, number of gradients = 33, axial plane, in-plane resolution = 0.8281 × 0.8281 mm). DTI images from all participants were collected from the same scanner and processed with the same software and parameters.

### DTI Processing

The DTI images were automatically pre-processed and segmented using MRICloud (Mori et al., 2016).^2^ The tensor reconstruction and quality control followed the pipeline of DTIStudio (Jiang et al., 2006).^3^ MRICloud is a web-based platform that uses a fully automated multiatlas image parcellation algorithm that combines the image transformation algorithm, Large Deformation Diffeometric Mapping, based on complementary contrasts (e.g., MD, RD, FA, and fiber orientation; Ceritoglu et al., 2009), and a likelihood fusion algorithm for DTI multiatlas mapping and parcellation (Tang et al., 2014). This generated 168 regions of interest (ROIs), from which DTI scalar metrics (3 eigenvalues) were extracted. The analyses focused on global and medial temporal FA and RD values. Global FA and RD values were calculated as the average of 8 major bilateral white matter tracts (fornix, cingulum, uncinate fasciculus, corona radiata, super longitudinal fasciculus, inferior frontal occipital fasciculus, corpus callosum, and posterior thalamic radiation, as used in prior work by this group; Soldan et al., 2022). Medial temporal FA and RD values were calculated as the average of 3 bilateral MTL white matter tracts (hippocampal cingulum, fornix, and uncinate fasciculus), which have previously been linked to episodic memory performance in the current cohort (Alm et al., 2020). The tracts included in each of these composites were chosen *a priori*, because they provide robust estimates of FA and RD using the automatic MRICloud pipeline across a broad range of ages and levels of neurodegeneration (Rezende et al., 2019). Composites were used to reduce the number of statistical comparisons, and because prior literature has not provided a clear indication of which specific tracts are related to measures of cognitive reserve or contribute to brain maintenance.

### Cognitive Reserve Composite Score

Level of cognitive reserve (CR) was operationalized by a composite score that included three measures commonly used as proxies of CR, hypothesized to reflect lifetime cognitive experiences: (1) years of education; (2) scores from the National Adult Reading Test (NART; Nelson, 1982); and (3) scores on the Wechsler Adult Intelligence Scale-Revised (WAIS-R) vocabulary subtest (Wechsler, 1981). Both the NART and the WAIS-R were collected at study baseline (i.e., between 1995–2005), when all participants were cognitively normal. These measures were standardized as z-scores then averaged to calculate the composite, given that they are highly correlated and load on a single factor in a factor analysis (for details, see Soldan et al., 2013). Prior studies have demonstrated this composite to be associated with better clinical and cognitive outcomes after adjusting for biomarkers of AD pathology (Soldan et al., 2013, 2015, 2017; Pettigrew et al., 2017).

### Vascular Risk Composite Score

Vascular risk factors were established through self-report during a medical history interview and available medical records. A composite vascular risk score was calculated using a previously validated approach (Gottesman et al., 2017) that sums 5 dichotomous vascular risk factors (each coded as 0 = absent, 1 = recent/remote): diabetes, hypertension, hypercholesterolemia, obesity (based on body mass index >30 kg/m^2^), and current smoking (i.e., within the past 30 days).

### APOE Genotype

APOE genotypes were determined by restriction endonuclease digestion of PCR-amplified genomic DNA (performed by Athena Diagnostics, Worcester, MA). Genotypes were coded dichotomously (*APOE*-ε4 carriers = 1, non-carriers = 0).

### Statistical Analyses

Group differences in demographic and descriptive statistics were assessed by Wilcoxon rank sum tests for continuous variables and chi-square tests for binary variables.

Longitudinal linear mixed effects models were used to examine the associations of the CR composite score with level of, and longitudinal change in, global and MTL FA and RD (see Supplementary Material 1). These models included linear effects of time and were specified with random intercepts and slopes (Laird and Ware, 1982; Diggle et al., 1994). Separate models were run for each DTI measure, which served as the dependent variable, including baseline measures and all available follow-up. Time was modeled as a continuous variable in the unit of year. All other continuous variables were standardized before model fitting. The primary set of models included the following predictors: time (years from baseline), sex, age at first DTI scan, diagnosis at time of scan (normal vs. MCI), CR composite score, and the interaction (cross-product) of each predictor with time. The main effect of the CR composite score, and the two-way CR composite score × time interaction, were of primary interest, as these terms reflect differences in levels and rates of change of the DTI measures (respectively) as a function of the CR composite score.

The second set of linear mixed regression models tested whether the relationship between the CR composite score and level of, and longitudinal change in, the DTI measures was modified by age, *APOE*-ε4 genetic status, and the vascular risk composite score, with separate models run for each of these variables of interest. This was accomplished by re-running the models above, this time including the additional relevant two-way and three-way interactions. For example, for the models assessing the impact of age, the CR composite score × age interaction indicates whether the association of the CR composite score and levels of the DTI measures differed by baseline age, whereas the three-way CR composite score × age × time interaction indicates whether the relationship between the CR composite score and rates of change in the DTI measures differed by baseline age.

Sensitivity analyses examined whether the patterns of results changed when individuals with MCI were excluded. Separate models were not run for the participants with MCI given the small sample size (*n* = 26). Estimates and *p*-values are reported, and *p*-values < 0.05 were considered significant. All analyses were run in R, version 3.5.0.

### Research Data

Anonymized BIOCARD study data are available upon request from qualified investigators; for more details, visit www.biocard-se.org.

## Results

### Study Sample Characteristics

Baseline descriptive characteristics of all participants included in the analyses, stratified by diagnosis, are reported in Table 1. Compared to those with normal cognition, individuals with MCI were older, less likely to be White, performed worse on the Mini-Mental State Examination, had lower CR composite scores, higher vascular risk scores, and lower global and MTL FA values, and higher global and MTL RD values (all *p* < 0.01). Additionally, the mean follow-up time between participants’ first and last DTI scan was less for participants with MCI. Of note, higher CR composite scores were correlated with higher MMSE scores across the non-demented participants, partialling out age and sex, [*r*(188) = 0.39, *p* < 0.01], and in the cognitively normal subgroup [*r*(162) = 0.26, *p* < 0.01].

**Table 1:** Baseline characteristics of participants included in the analyses. Values reflect mean (SD) unless otherwise indicated.

**Variable**	**All subjects in analysis *N* = 192**	**Cognitively normal at baseline *N* = 166**	**MCI at baseline *N* = 26**
Age at baseline DTI scan	70.2 (8.50)	69.5 (8.17)	74.4 (9.53)^*^
Sex female, *N* (%)	120 (62.5%)	105 (63.3%)	15 (57.7%)
White race, *N* (%)	186 (96.9%)	164 (98.8%)	22 (84.6%)^*^
APOE4 carriers, *N* (%)	62 (32.3%)	54 (32.5%)	8 (30.8%)
Years of education	17.3 (2.29)	17.3 (2.22)	17.1 (2.72)
MMSE at baseline	29.0 (1.24)	29.3 (0.93)	27.3 (1.57)^*^
CR composite score	0.13 (0.78)	0.21 (0.70)	−0.37 (1.04)^*^
Global FA	0.47 (0.02)	0.47 (0.02)	0.45 (0.02)^*^
MTL FA	0.41 (0.02)	0.41 (0.02)	0.39 (0.02)^*^
Global RD	7.0^−4^ (5.10^−5^)	6.9^−4^ (4.85^−5^)	7.3^−4^ (5.47^−5^)^*^
MTL RD	9.3^−4^ (7.99^−5^)	9.2^−4^ (7.58^−5^)	1.0^−3^ (7.44^−5^)^*^
Number of DTI scans over time [range]	2.2 (0.73) [1–3]	2.3 (0.72) [1–3]	1.9 (0.73) [1–3]
Time between first and last DTI scan, in years [range]	2.5 (1.49) [0–4.7]	2.7 (1.46) [0–4.7]	1.8 (1.51) [0–4.2]^*^
Composite vascular risk score	1.4 (1.09)	1.3 (1.06)	2.0 (1.08)^*^

* Significant difference between groups, *p* < 0.01, using Wilcoxon rank sum tests for continuous variables and chi-square tests for dichotomous variables.

CR, cognitive reserve. DTI, diffusion tensor imaging. FA, fractional anisotropy. MCI, mild cognitive impairment. MMSE, Mini-Mental State Examination (Folstein et al., 1975); MTL, medial temporal lobe; RD, radial diffusivity.

### Association of Baseline CR With Level of and Change in DTI Measures

In both the full sample (*n* = 192) and the subset with normal cognition (*n* = 166), there were significant effects of time for all DTI measures, indicated by decreases in FA values and increases in RD values over time, in both the global and MTL DTI composites (all *p* < 0.001). However, there were no significant CR × time interactions in either group (all *p* > 0.14), indicating that the rate of change in DTI measures over time does not differ by level of CR. Additionally, in reduced models that excluded the non-significant CR × time interaction terms, there were no main effects of CR on global or MTL FA or RD in the full sample (all *p* > 0.11). Among the subset with normal cognition, there was a significant main effect of CR on MTL RD only [estimate = −0.141, 95% CI (−0.244, −0.039), *p* = 0.007; all other *p* > 0.08], indicating that higher CR composite scores were associated with lower RD in the MTL tracts.

For the model covariates (from the reduced models that excluded the non-significant interaction terms), in the full sample, higher age at baseline was associated with lower FA and higher RD values in the global and MTL DTI composites (all *p* < 0.001), and female sex was associated with lower global and MTL RD values (both *p* < 0.04). Furthermore, an MCI diagnosis was associated with lower global and MTL FA values (both *p <* 0.04), as well as higher global and MTL RD values (both *p* < 0.03). In addition, the rates of change in global and MTL FA values, and in global RD values, over time were greater among older participants, as indicated by significant age × time interactions (both *p* < 0.05), with a similar trend for MTL RD values (*p* = 0.051). The patterns of covariate effects were similar for the subset with normal cognition, except that the age × time interactions were only significant for the global RD values (*p* = 0.009), whereas these interactions were trending for the global and MTL FA values and MTL RD values (all *p* ≤ 0.10).

### Interaction Between CR and Age on DTI Measures

**Figure 1:**
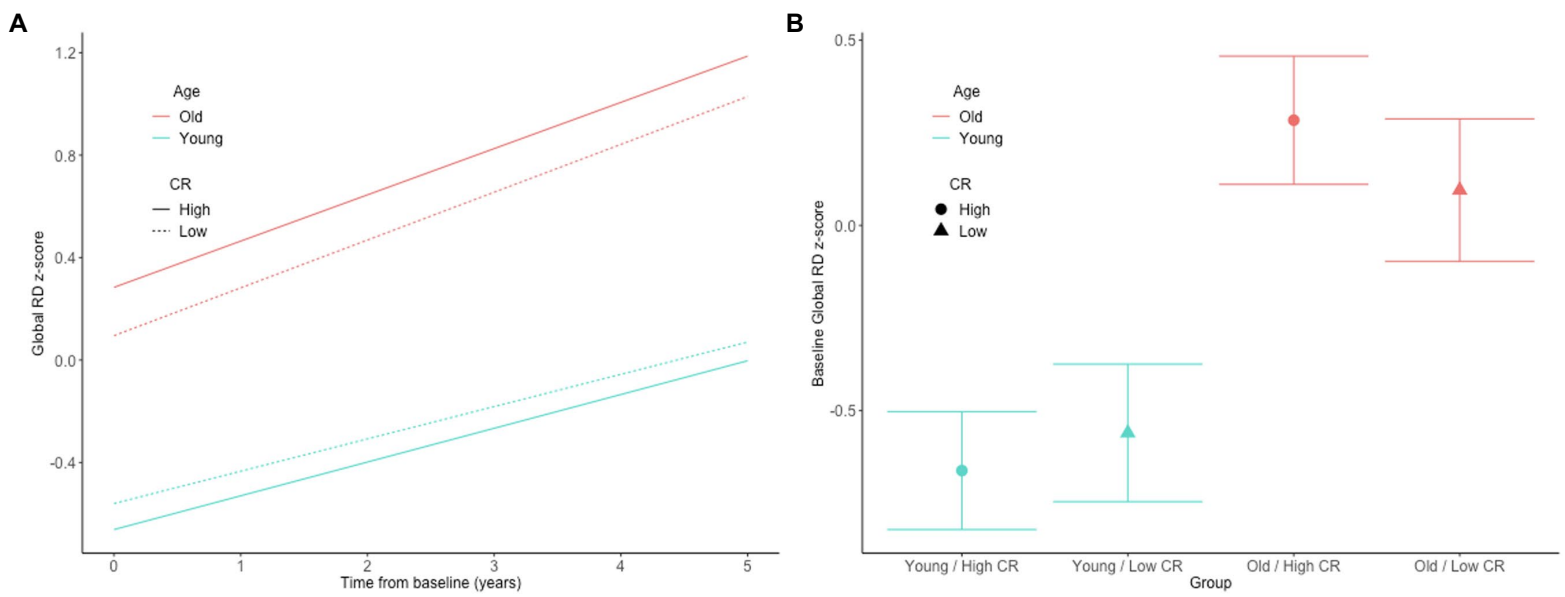
Estimates of the association between CR composite scores and age in relationship to longitudinal change in **(A)** and baseline level of **(B)** white matter microstructure, as measured by global RD in the full, non-demented sample. The CR composite score and age were dichotomized by median split for illustration purposes, but treated as continuous variables in the linear mixed effects models.

**Figure 2:**
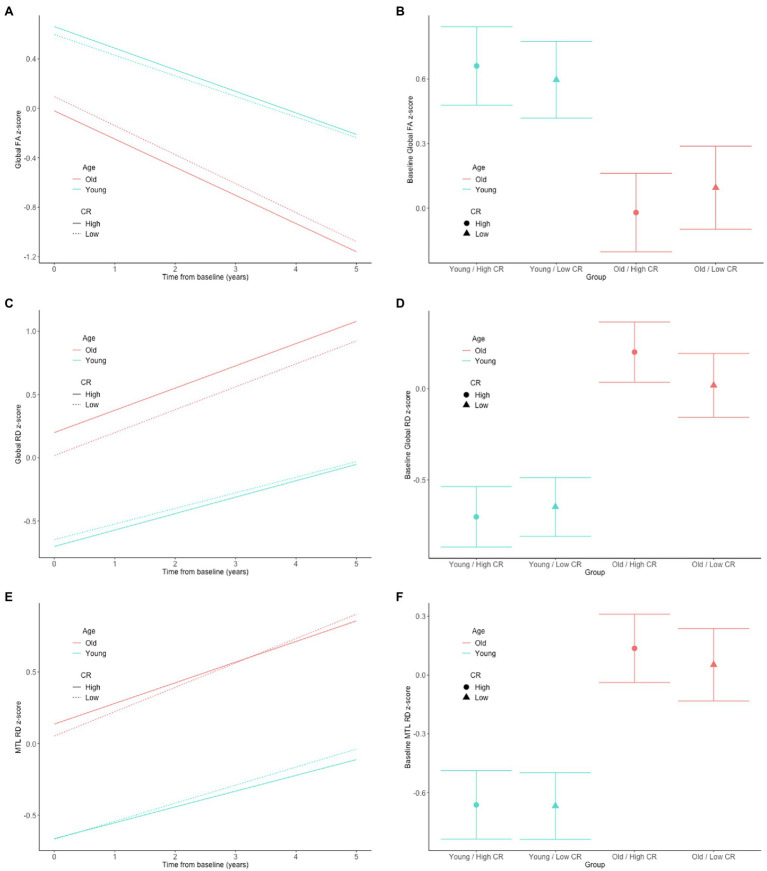
Estimates of the association between CR composite scores and age in relationship to longitudinal change in (left) and baseline level of (right) white matter microstructure, as measured by global FA **(A,B)**, global RD **(C,D)**, and MTL RD **(E,F)** in the cognitively normal sample. The CR composite score and age were dichotomized by median split for illustration purposes, but treated as continuous variables in the linear mixed effects models.

In both the full sample (*n* = 192) and the subset with normal cognition (*n* = 166), there were no significant CR × age × time interactions for any of the DTI measures (all *p* > 0.63), indicating that the associations between the CR composite score and rates of change in the DTI measures over time do not differ by baseline age. The results for the reduced models, which excluded the non-significant CR × age × time interaction terms, are shown in Table 2. In the full sample, there was a CR × age interaction for global RD (Figures 1A,B). Additionally, among the subset with normal cognition, there were significant CR × age interactions for global FA (Figures 2A,B), global RD (Figures 2C,D), and MTL RD (Figures 2E,F). For both groups, these CR × age interactions indicate that the relationship of the CR composite score to baseline levels of the DTI measures differed for individuals of younger compared to older baseline ages. Specifically, among younger adults, higher CR composite scores were associated with higher FA values and lower RD values at baseline; in contrast, among older adults, higher CR composite scores were associated with lower FA values and higher RD values at baseline. Note that while age and CR were dichotomized for illustration purposes, they were modeled as continuous variables in the statistical analyses.

**Table 2:** Results of the reduced longitudinal mixed effects models examining whether the relationship between the CR composite score and white matter microstructure differs by baseline age (see text for details).

Model Predictors	Global FA		MTL FA		Global RD		MTL RD	
	Estimate (95% CI)	*p*-value	Estimate (95% CI)	*p*-value	Estimate (95% CI)	*p*-value	Estimate (95% CI)	*p*-value
**Full Sample**								
CR Composite	0.017 (−0.104, 0.137)	0.788	−0.006 (−0.129, 0.115)	0.918	−0.037 (−0.146, 0.072)	0.505	−0.051 (−0.161, 0.059)	0.369
CR Composite × age	−0.1 (−0.204, 0.004)	0.064	−0.063 (−0.164, 0.039)	0.232	**0.127 (0.031, 0.224)**	**0.011** ^*^	0.067 (−0.029, 0.162)	0.178
CR Composite × time	0.008 (−0.033, 0.049)	0.717	0.028 (−0.014, 0.071)	0.19	−0.002 (−0.032, 0.027)	0.888	−0.021 (−0.049, 0.008)	0.159
**Cognitively normal**
CR Composite	0.084 (−0.047, 0.215)	0.214	0.04 (−0.098, 0.177)	0.577	−0.092 (−0.21, 0.027)	0.134	−0.096 (−0.221, 0.029)	0.137
CR Composite × age	**−0.161 (−0.289, −0.033)**	**0.015** ^*^	−0.092 (−0.22, 0.037)	0.167	**0.211 (0.093, 0.33)**	**0.001** ^*^	**0.127 (0.004, 0.249)**	**0.047** ^*^
CR Composite × time	0.006 (−0.039, 0.051)	0.787	0.026 (−0.021, 0.072)	0.287	−0.006 (−0.039, 0.026)	0.708	−0.02 (−0.051, 0.01)	0.201

*p < 0.05.

When the models were restricted to the *n* = 155 individuals who were cognitively normal at *both* their first and last DTI scan, the CR × age interactions for global FA (estimate = −0.163, 95% CI (−0.289, −0.036), *p =* 0.013), global RD (estimate = 0.215, 95% CI (0.099, 0.33), *p* < 0.001), and MTL RD (estimate = 0.124, 95% CI (0.003, 0.246), *p =* 0.05) remained significant.

In exploratory analyses, there were similar CR × age interactions for the majority of the individual white matter tracts included in the global composite (see Supplementary Table S1), suggesting these effects are quite widespread, rather than driven by tract-specific effects. Consistent with the results of the global composite score, none of the CR × age × time interactions were significant (all *p* > 0.10, data not shown).

### Interaction Between CR and *APOE*-**ε**4 Genotype Status on DTI Measures

In both the full sample (*n* = 192) and the subset with normal cognition (*n* = 166), there were no significant CR × *APOE*-ε4 × time interactions for any of the DTI measures (all *p* > 0.33); similarly, in the reduced models that excluded these non-significant 3-way interaction terms, there were no significant CR × *APOE*-ε4 interactions (all *p* > 0.35). This indicates that the associations between the CR composite score and level of, and rate of change in the DTI measures over time, did not differ by *APOE*-ε4 genotype status. Additionally, *APOE*-ε4 genotype status was unrelated to the levels of (all *p* > 0.09), and rate of change over time in (all *p* > 0.07), the DTI measures.

### Interaction Between CR and Vascular Risk on DTI Measures

In both the full sample (*n* = 192) and the subset with normal cognition (*n* = 166), there were no significant CR × vascular risk score × time interactions for any of the DTI measures (all *p* > 0.57); similarly, in the reduced models that excluded these non-significant 3-way interaction terms, there were no significant CR × vascular risk score interactions (all *p* > 0.68). This indicates that the associations between the CR composite score and level of, and rate of change in the DTI measures over time, did not differ by level of vascular risk. Additionally, the vascular risk score was unrelated to the levels of (all *p* > 0.10), and rate of change in (all *p* > 0.38), the DTI measures.

## Discussion

This study examined whether lifetime experiences, as measured by a CR composite score, are related to the level of, and short-term rate of change in white matter microstructural integrity, as measured by global and MTL FA and RD values derived from DTI. The primary finding is that the relationship between the CR composite score and white matter microstructure differed by baseline age, such that among participants with younger baseline ages, higher CR composite scores tended to be associated with higher FA and lower RD scores, whereas among older participants, higher CR scores were related to lower FA and higher RD scores. Furthermore, the relationship between the CR composite score and white matter microstructure was not modified by *APOE*-ε4 genetic status or level of vascular risk. Taken together, these results suggest that among individuals in late middle age, greater educational and intellectual attainment may be associated with better global white matter microstructure, reflecting neuroprotective effects of these lifetime experiences. In contrast, among older adults, these same experiences are associated with worse white matter microstructural integrity, potentially reflecting a greater ability to tolerate or compensate for age and/or disease-related declines in white matter integrity among those with higher than lower CR scores, as discussed in greater detail below.

To our knowledge, this is the first study to evaluate the relationship between CR proxies and DTI-derived brain white matter microstructure over time. Consistent with prior literature, we found that global and MTL white matter microstructure decreased over time, particularly among older participants (e.g., Bennett and Madden, 2014; Bender et al., 2016). However, we found no evidence of an association between lifetime experiences, as measured by a CR composite score, and short-term rate of change in global or MTL white matter microstructure, and no evidence that the CR proxy score reduced age-related changes in the white matter microstructure over time. Although these results provide preliminary evidence suggesting that lifetime experiences do not slow or prevent short-term changes in white matter microstructure among older adults, future studies including a larger array of CR proxies, and longer DTI follow-up, are needed to replicate these findings.

Our finding of an age-dependent relationship between the CR proxy score and white matter microstructural measures may help explain the inconsistencies in the literature, as prior studies to date have been quite mixed (as described above). For example, a large cross-sectional study (*N* > 600) found that greater occupational complexity in young to middle-adulthood was associated with higher FA globally and in all four lobes 10–15 years later, when participants were in middle age (Kaup et al., 2018). This is consistent with our finding of a more positive relationship between higher educational and intellectual achievement and higher global FA values among participants with lower baseline ages. Furthermore, in the present study, the interactions between the CR composite score and baseline age were particularly evident among the participants with normal cognition (relative to the full non-demented sample) and remained significant in the subset of individuals who were cognitively normal at all of their DTI time points. This may suggest that the neuroprotective effects of certain lifetime experiences on white matter microstructure decline after middle age, potentially because the beneficial effects of these lifetime experiences on white matter microstructure are smaller than age and disease-related changes, and are eventually overwhelmed by the latter. Consistent with this hypothesis, the estimated age-related difference in the level and rate of change in both FA and RD was greater than the CR-related difference in these measures (see Figures 1, 2 for an illustration).

Our results are also broadly consistent with a prior study from our cohort, which reported that participants with higher CR composite scores had lower white matter hyperintensity levels when they were largely in late middle age (Pettigrew et al., 2020), providing evidence for a beneficial effect of educational and intellectual attainment on aspects of white matter structure. Furthermore, although in both studies the CR composite score was not related to the short-term rate of change in the white matter structural measures, the finding that participants with higher CR proxy scores (and lower baseline ages) had more intact white matter may be consistent with the concept of brain maintenance. Specifically, given that baseline measures in white matter indices were already different among participants with higher vs. lower CR scores may indicate that these white matter measures were influenced by educational and intellectual attainment at an earlier time point, and that these lifetime measures may act over longer time periods. Future studies that examine structural brain changes among middle-aged participants over longer time periods are needed to further test this hypothesis.

The finding that among participants with higher baseline ages, higher CR scores tended to be associated with reduced white matter integrity, as measured by both FA and RD, is consistent with prior studies reporting negative associations between level of education and FA values among cognitively normal and MCI participants (Arenaza-Urquijo et al., 2011; Vaqué-Alcázar et al., 2017). These results may suggest that while neuroprotective effects of higher educational and intellectual attainment on white matter microstructure are not measurable at older ages, these experiences instead allow individuals to tolerate greater amounts of age and/or disease-related changes in white matter microstructure, while maintaining cognitive performance over time. It is noteworthy that, although the impact of white matter microstructural measures on cognitive performance were not examined in this study, higher CR composite scores were correlated with better global cognitive performance, as measured by MMSE scores. This suggests that despite potentially having worse white matter microstructure, participants with higher educational and intellectual attainment tended to perform better cognitively. Taken together with the lack of association between the CR composite score and short-term rate of change in white matter microstructure over time (described above), the results from participants with older baseline ages are more consistent with the concept of cognitive reserve, rather than brain maintenance.

Although not examined in the present study, a possible mechanism by which higher educational or intellectual attainment may allow individuals to compensate for age or disease-related structural brain changes is by fostering functional network connectivity. For example, prior resting-state functional MRI (fMRI) studies among cognitively normal older adults have linked higher educational attainment (Arenaza-Urquijo et al., 2013; Perry et al., 2017), as well as greater engagement in cognitively stimulating leisure activities (Soldan et al., 2021) to greater functional network connectivity and improved cognitive performance. Additionally, task-based fMRI studies have provided evidence that older participants with high CR may have more efficient functional brain networks or may be able to rely on alternate or compensatory functional networks in the presence of neurodegenerative changes (Steffener et al., 2011; Franzmeier et al., 2017; Stern et al., 2018). This interpretation is also consistent with the “less wiring, more firing hypothesis,” whereby increased functional activity of intact pathways may offset declines in white matter structural integrity, such as those measured by DTI (Daselaar et al., 2015). Enhancements in functional connectivity (e.g., greater network efficiency, capacity, or flexibility; Barulli and Stern, 2013) may support maintained cognitive performance in the presence of age or disease-related brain changes, and be one mechanism by which educational and intellectual attainment influence cognitive and clinical outcomes.

Exploratory analyses further suggested that the interaction between age and CR on white matter microstructure was evident in the majority of the individual tracts included in the composite measures. Conceptually, it seems reasonable that fairly broad measures of lifetime experience (i.e., education and literacy levels) would have global effects on the brain, as opposed to highly localized effects. However, future studies are needed to replicate this finding.

In this study, we found no evidence that the relationship between the CR composite score and level of, or rate of change in white matter microstructural integrity was modified by *APOE*-ε4 genotype. Additionally, *APOE*-ε4 genotype was unrelated to the level of, or rate of change in, global or MTL white matter microstructure, which is consistent with prior studies among cognitively normal older adults (Honea et al., 2009; Heise et al., 2011; Patel et al., 2013). As suggested by prior literature, *APOE*-ε4 has a greater impact on AD pathology, particularly amyloid burden (Morris et al., 2010; Resnick et al., 2015; Toledo et al., 2015; Pletnikova et al., 2018; Baek et al., 2020), rather than directly on white matter integrity, although we cannot rule out the possibility that *APOE*-ε4 affects axonal and synaptic integrity in ways that are not measurable through current DTI technologies (Adalbert et al., 2007).

We also found that vascular risk, as measured by a composite score, did not modify the relationship between CR and white matter microstructure. This suggests similar relationships between educational and intellectual attainment and white matter microstructure among individuals with higher and lower levels of vascular risk. However, the usage of a composite score precludes us from concluding that results would be the same for individual vascular risk factors. Additionally, the vascular risk composite score was unrelated to DTI white matter microstructure. This latter result appears inconsistent with prior studies that have found relationships between higher vascular risk (including hypertension, obesity, and diabetes) and poorer white matter integrity, as indexed by lower FA among middle-aged and older adults (e.g., Falvey et al., 2013; Zhang et al., 2018; Ingo et al., 2021). These null results may be attributed to somewhat lower levels of vascular risk among participants in the present study compared to other cohorts, as well as the high level of education among our participants that may enable them to more effectively manage their vascular conditions through medications.

Although several studies have examined the relationship of lifetime experiences to white matter microstructure, the neurobiological mechanisms underlying these associations are not well understood. Prior research has predominantly focused on the role of brain-derived neurotrophic factor (BDNF) as one possible mechanism. For example, prior studies in rodent models have demonstrated that BDNF is upregulated in brain regions including the hippocampus among animals exposed to enriched environments that provide more cognitive and physical stimulation (Ickes et al., 2000). BDNF is known to support neurogenesis, as well as synaptic plasticity, such as by enhancing synaptic vesicle movement and docking or increasing long-term potentiation, which is important for learning and memory (Pozzo-Miller et al., 1999; Rossi et al., 2006). Animal models have also shown that oligodendrocytes, which are myelinating cells that wrap around axons, promote BDNF production in the spinal cord and brain (Dougherty et al., 2000; Bradl and Lassmann, 2010; Ramos-Cejudo et al., 2015). This suggests that higher BDNF levels may help to preserve white matter microstructure/myelination. In an effort to extend these findings to humans, Ward et al. (2015) proposed that the *BDNF* Val66Met polymorphism interacts with CR to enhance executive function (Ward et al., 2015). Moreover, Collins and colleagues found that early-life education was associated with increased serum levels of BDNF in old age (Collins et al., 2021). Although much less is known about variables that regulate BDNF levels in humans, these findings suggest that early-life enrichment experiences, such as education, as well as lifelong experiences that promote literacy, may enhance the production of BDNF or other neurotrophic factors, thereby helping to maintain white matter microstructure across the lifespan. Lifetime experiences may also alter synaptic architecture or neurotransmitter transmission to promote resilience against disease-related pathology (Arnold et al., 2013; Garibotto et al., 2013; Robertson, 2013; Boros et al., 2017). Future studies are needed to further address these possibilities.

This study has limitations. First, DTI provides only an indirect measure of white matter integrity (Jones et al., 2013). Thus, the results of this study are limited by the use of this technique, and future studies could expand this work by using DTI in conjunction with other measures of white matter integrity (Bennett and Madden, 2014). Furthermore, this study evaluated FA and RD composites to minimize the number of multiple comparisons, though future studies might also evaluate other DTI measures, such as axial diffusivity. The BIOCARD cohort is highly educated, primarily White, and by design has a strong family history of AD, therefore limiting the generalizability of these results. Additionally, we focused on a single measure of lifetime experiences—a CR proxy score—and therefore cannot address the extent to which other CR proxies, such as occupation or bilingualism, differentially influence white matter microstructure. Finally, on average, there were only 2.5 years between an individual’s first and last DTI scan, which is a brief snapshot of brain changes that may occur over decades. It is possible, therefore, that results may differ over longer follow-up periods.

## Data Availability Statement

Anonymized data used in the analyses presented in this report are available on request from qualified investigators (www.biocard-se.org).

## Ethics Statement

The studies involving human participants were reviewed and approved by Johns Hopkins University Institutional Review Board. The patients/participants provided their written informed consent to participate in this study.

## Author Contributions

MA, AS, and CP contributed to the study design. RB, AS, and CP drafted the manuscript and interpreted the data. YZ performed the statistical analyses and contributed to the interpretation of the data. AF contributed to processing the MRI scans. All authors revised the manuscript for content. All authors contributed to the article and approved the submitted version.

## Funding

This work was supported by grants from the National Institutes of Health (U19-AG033655 and P30-AG066507).

## Conflict of Interest

MA is an advisor to Eli Lily.

The remaining authors declare that the research was conducted in the absence of any commercial or financial relationships that could be construed as a potential conflict of interest.

## Publisher’s Note

All claims expressed in this article are solely those of the authors and do not necessarily represent those of their affiliated organizations, or those of the publisher, the editors and the reviewers. Any product that may be evaluated in this article, or claim that may be made by its manufacturer, is not guaranteed or endorsed by the publisher.

## Footnotes

^1^Reserve and Resilience (2021). Framework for terms used in research of reserve and resilience. Available at: https://reserveandresilience.com/framework/ (Accessed October 18, 2021).

^2^ https://braingps.mricloud.org

^3^ www.MRIStudio.org
