# Supplementary material for: Age-Dependent Association Between Cognitive Reserve Proxy and Longitudinal White Matter Microstructure in Older Adults
Source: Front Psychol. 2022 Jun 10;13:859826. doi: 10.3389/fpsyg.2022.859826 (PMC9226781; doi:10.3389/fpsyg.2022.859826)
Supplement: Supplementary file 1 [file Data_Sheet_1.PDF]

Supplementary Materials

Supplementary Materials 1: Formula for the longitudinal linear mixed effects models.

Let Y_{ij} denote the outcome variable for the ith participant at the jth visit. Let Z_{ij} be the time since baseline and let X_{ij} denote all other variables including all interaction terms. We fit the following models:

$$Y_{ij} = \beta_0 + X_{ij}^T \cdot \beta_1 + Z_{ij} \cdot \beta_2 + \alpha_{i0} + Z_{ij} \cdot \alpha_{i1} + \epsilon_{ij},$$

Where β_0 is the fixed effect intercept, β_1 is the fixed effect covariate effect, β_2 is the time slope, α_{i0} is the random intercept that varies across participants, α_{i1} is the random time slope that varies across participants, and ϵ_{ij} is independently and identically distributed error terms across participants and visits. We assume that $(\alpha_{i0}, \alpha_{i1})$ follows multivariate normal distribution with mean zeros and variance-covariance matrix Σ and that ϵ_{ij} follows normal distribution with mean zero and variance σ^2.

<u>Table S1</u>. Results of the reduced longitudinal mixed effects models examining whether the relationship between the CR composite score and baseline white matter microstructure differs by baseline age in individual white matter tracts. The results below show the estimate (95% CI) and *p*-values for the CR x age interaction terms.

Model Predictor	**Full Sample**		**Cognitively normal**			
	RD		FA		RD	
	Estimate (95% CI)	*p*-value	Estimate (95% CI)	*p*-value	Estimate (95% CI)	*p*-value
Posterior Thalamic Radiation	0.176 (0.064, 0.289)	**0.003 ***	-0.167 (-0.305, -0.029)	**0.02 ***	0.249 (0.108, 0.391)	**0.001 ***
Anterior Corona Radiata	0.112 (0.010, 0.215)	**0.04 ***	-0.163 (-0.298, -0.028)	**0.02 ***	0.182 (0.051, 0.312)	**0.008 ***
Superior Corona Radiata	0.158 (0.054, 0.263)	**0.004 ***	-0.130 (-0.273, 0.012)	0.08 ^	0.226 (0.094, 0.357)	**0.001 ***
Posterior Corona Radiata	0.164 (0.053, 0.276)	**0.005 ***	-0.025 (-0.183, 0.133)	0.76	0.220 (0.075, 0.365)	**0.004 ***
Cingulum	0.077 (-0.035, 0.189)	0.18	-0.102 (-0.241, 0.037)	0.16	0.120 (-0.015, 0.255)	0.09 ^
Hippocampal Cingulum	0.109 (-0.001, 0.219)	0.06 ^	-0.045 (-0.191, 0.101)	0.55	0.146 (0.013, 0.278)	**0.03 ***
Superior Longitudinal Fasciculus	0.124 (0.011, 0.237)	**0.03 ***	-0.152 (-0.297, -0.006)	**0.04 ***	0.213 (0.071, 0.355)	**0.004 ***
Inferior Frontal Occipital Fasciculus	0.077 (-0.030, 0.185)	0.17	-0.154 (-0.297, -0.010)	**0.04 ***	0.155 (0.023, 0.287)	**0.02 ***
Uncinate Fasciculus	0.091 (-0.013, 0.196)	0.09 ^	-0.088 (-0.238, 0.062)	0.26	0.173 (0.039, 0.307)	**0.01 ***
Fornix	0.031 (-0.068, 0.130)	0.55	-0.075 (-0.203, 0.053)	0.25	0.075 (-0.055, 0.204)	0.26
Genu Corpus Callosum	0.072 (-0.039, 0.181)	0.21	-0.128 (-0.262, 0.006)	0.07 ^	0.139 (0.011, 0.266)	**0.04 ***
Body Corpus Callosum	0.126 (0.015, 0.236)	**0.03 ***	-0.197 (-0.345, -0.050)	**0.01 ***	0.213 (0.077, 0.349)	**0.003 ***
Splenium Corpus Callosum	0.153 (0.047, 0.260)	**0.006 ***	-0.185 (-0.324, -0.046)	**0.01 ***	0.250 (0.118, 0.382)	**< 0.001 ***

* $p < 0.05$; ^ $p < 0.10$
